# Quantitative susceptibility mapping in ischemic stroke patients after successful recanalization

**DOI:** 10.1038/s41598-021-95265-3

**Published:** 2021-08-06

**Authors:** Jasmin Probst, Marco Rohner, Malin Zahn, Marco Piccirelli, Athina Pangalu, Andreas Luft, Andreas Deistung, Jan Klohs, Susanne Wegener

**Affiliations:** 1 Department of Neurology, Clinical Neuroscience Center, University Hospital Zurich and University of Zurich, Frauenklinikstrasse 26, 8091 Zurich, Switzerland; 2 Department of Neuroradiology, Clinical Neuroscience Center, University Hospital Zurich, Zurich, Switzerland; 3 University Clinic and Outpatient Clinic for Radiology, University Hospital Halle (Saale), Halle, Germany; 4 Institute for Biomedical Engineering, University of Zurich and ETH Zurich, Zurich, Switzerland; 5 Cereneo Center for Neurology and Rehabilitation, Vitznau, Switzerland

**Keywords:** Central nervous system, Brain, Predictive markers, Cerebrovascular disorders, Stroke, Stroke, Outcomes research, Translational research

## Abstract

Quantitative susceptibility mapping (QSM) is a novel processing method for gradient-echo magnetic resonance imaging (MRI). Higher magnetic susceptibility in cortical veins have been observed on susceptibility maps in the ischemic hemisphere of stroke patients, indicating an increased oxygen extraction fraction (OEF). Our goal was to investigate susceptibility in veins of stroke patients after successful recanalization in order to analyze the value of QSM in predicting tissue prognosis and clinical outcome. We analyzed MR images of 23 patients with stroke due to unilateral middle cerebral artery (MCA)-M1/M2 occlusion acquired 24–72 h after successful thrombectomy. The susceptibilities of veins were obtained from QSM and compared between the stroke territory, the ipsilateral non-ischemic MCA territory and the contralateral MCA territory. As outcome variables, early infarct size and functional disability (modified Rankin Scale, mRS) after 3–5 months was used. The median susceptibility value of cortical veins in the ischemic core was 41% lower compared to the ipsilateral non-ischemic MCA territory and 38% lower than on the contralateral MCA territory. Strikingly, in none of the patients prominent vessels with high susceptibility signal were found after recanalization. Venous susceptibility values within the infarct did not correlate with infarct volume or functional disability after 3–5 months. Low venous susceptibility within the infarct core after successful recanalization of the occluded vessel likely indicates poor oxygen extraction arising from tissue damage. We did not identify peri-infarct tissue with increased susceptibility values as potential surrogate of former penumbral areas. We found no correlation of QSM parameters with infarct size or outcome.

## Introduction

Magnetic susceptibility is a fundamental physical property of materials that describes their ability to become magnetized. It represents a unique contrast mechanism to image tissue in magnetic resonance imaging (MRI)^1–3^. Susceptibility-based tissue contrasts can be deduced by post-processing of the complex-valued signal derived from T2*-weighted gradient-echo scans^4–9^. While the technique susceptibility weighted imaging (SWI) has already been established in neuroradiological diagnostics, its successor quantitative susceptibility mapping (QSM) is increasingly gaining consideration in clinical research. Both techniques reveal anatomic and physiologic information about the type of tissue and offer superb visualizations of the cerebral venous vasculature^1,10–12^. As susceptibility reflects the magnetizability of a tissue, it is sensitive to local and global oxygenation. The oxygen extraction fraction (OEF) level in cerebral veins directly relates to the concentration of paramagnetic deoxygenated hemoglobin molecules in the vessels^12–14^. Thus, magnetic susceptibility based contrast have been used to describe oxygenation status in the brain^14,15^.

QSM has been applied in the assessment of vessel function and oxygen metabolism in patients and animal models with acute ischemic stroke^16–19^. In patients with acute stroke, prominent vessels with high magnetic susceptibility were observed with SWI and QSM^18,20–23^. These have been referred to as asymmetrically prominent cortical veins (APCVs) and were found within the infarcted perfused area, indicating tendency of clinical deterioration in stroke patients^18^. The appearance of prominent vessels on QSM has been associated with the ischemic penumbra (or “tissue at risk”) which is characterized by an increase in OEF (misery perfusion) and good tissue prognosis after recanalization^18–20^. In penumbral areas, increased OEF likely leads to higher deoxyhemoglobin concentrations in cortical veins, which then increases magnetic susceptibility^19,22,24^. Animal models of transient focal ischemia have shown that prominent vessels persist long after recanalization^5,8^. However, susceptibility values in cortical veins have so far not been assessed in stroke patients after successfull recanalization.

The primary goal of this study was to extend previous preliminary findings with QSM to investigate the differences in susceptibility values of cortical veins between the infarcted area, border and non-infarcted areas in ischemic stroke patients after successful recanalization. We hypothesized that QSM could provide a measure of tissue oxygen extraction and thus potentially aid in discriminating tissues with different fate^19–21^. In addition, we correlated susceptibility values of cortical veins with functional disability after 3–5 months as well as infarct volume, an established predictive factor of clinical outcome in patients with middle cerebral artery (MCA) occlusions^25^.

## Materials and methods

### Study design and cohort description

During the period of December 2016 until October 2018, we screened MRI and clinical data of patients with ischemic stroke due to MCA-M1 or MCA-M2 occlusion referred for thrombectomy at the Department of Neurology, University Hospital Zurich. Post-treatment MR images were acquired 24–72 h after successful recanalization. Of 263 patients receiving thrombectomy for acute ischemic stroke in 39 cases MCA-M1 or MCA-M2 occlusion was present and T_2_*w raw data for QSM analysis could be extracted on site during official working times.

The study was approved by the local ethics committee (Kantonale Ethikkommission Zurich; KEK-ZH-Nr 2014-0304)**.** Informed consent was obtained from all participants as required by the ethics protocol. Only cases with successful recanalization verified by catheter angiography (defined as Thrombolysis in Cerebral Infarction (TICI) 2b or TICI 3 score^26^) were included in the study and if the recorded MRI data were of sufficient quality. To further evaluate recanalization, we analyzed flow in the MCA-M1 segment derived from routine transcranial duplex sonography (in cm/s) performed on day 0 to day 1 after thrombectomy.

**Figure 1:**
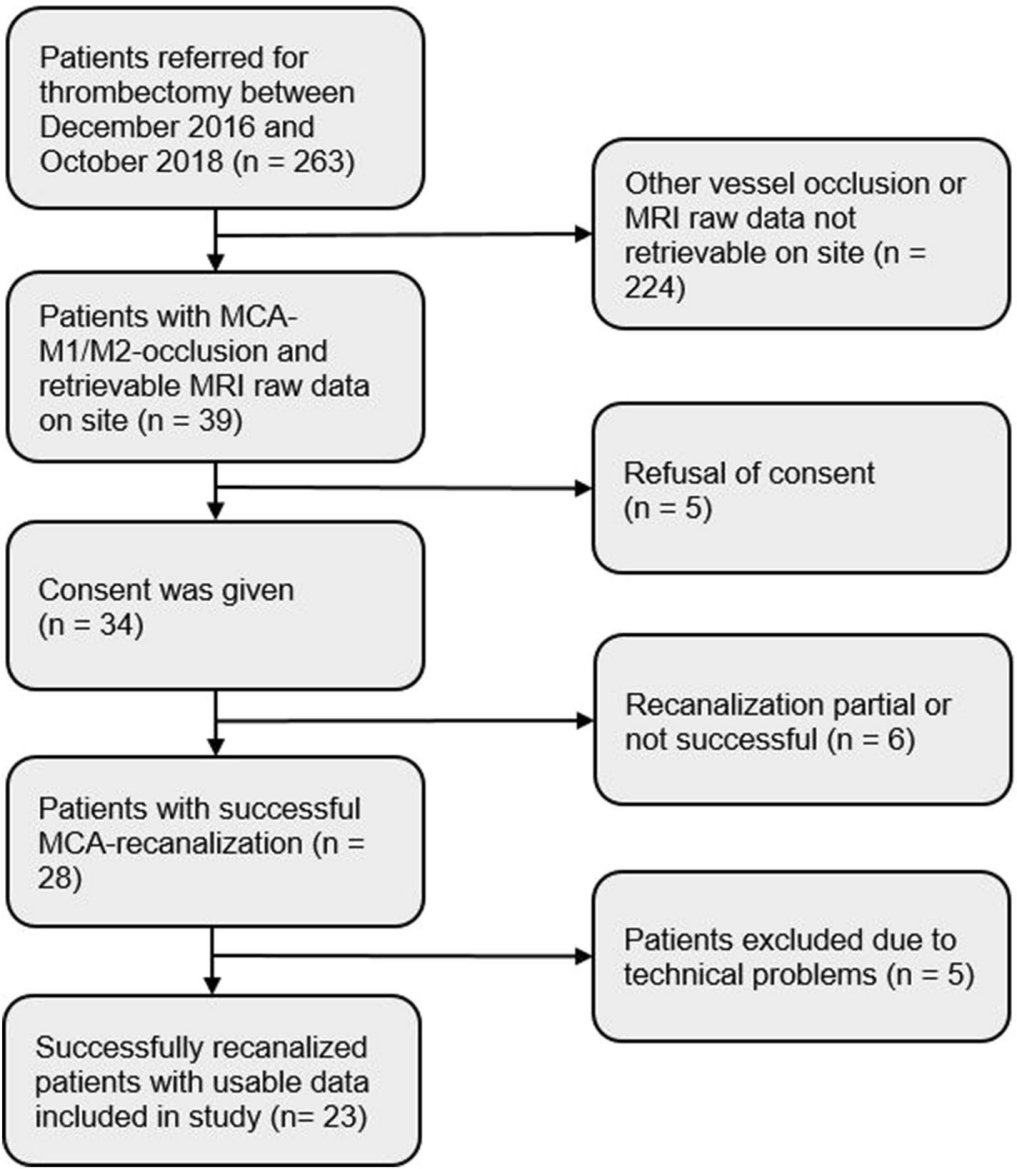
Patient inclusion criteria flow chart. Out of 263 patients referred for thrombectomy between December 2016 and October 2018 in 39 cases with MCA-M1/M2 occlusion MRI raw data for QSM analysis could be retrieved on site and assessed for eligibility. Patients were excluded due to refusal of consent, unsuccessful recanalization or due to technical problems that occurred during image analysis/processing (e.g. blurred images, massive hemorrhages or missing MRI-sequences essential to analysis) leading to 23 patients with successfully recanalized MCA-M1/M2 occlusion included in the current study.

Out of 39 patients eligible for further analysis, 34 agreed to take part in the data collection for this study. Six patients without or only partially successful recanalization according to the TICI reperfusion grade and five patients with technical issues (one with blurred images, one with missing MRI sequences essential to analysis, and three with massive hemorrhages compromising cortical vein identification) were excluded from further analysis, leading to 23 patients with successfully recanalized MCA-M1/M2 occlusion included in the current study. A flowchart illustrating patient selection is shown in Fig. 1.

Clinical variables including demographic data, medical history, associated risk factors and stroke etiology were gathered. In addition, information on treatment and outcome was collected, including information on intravenous thrombolysis, onset-to-needle time (ONT), NIH-Stroke Scale (NIHSS) on admission and after 24 h, and modified Rankin Scale (mRS) after 3–5 months. We determined infarct volume after recanalization from diffusion weighted imaging (DWI) which was performed between days 1 and 3 after stroke.

### MRI acquisition and post-processing

All MR images were acquired on a 3 Tesla MRI Scanner (Skyra, release VD13, Siemens Healthcare, Erlangen, Germany). DWI (acquisition type 2D, read-out segmented echo planar imaging approach, field of view 220 × 220 [mm^2^], number of slices 38, voxel size 1.1 × 1.1 × 3.0 [mm^3^], slice gap 0.9 [mm], repetition time (TR) 7340 [ms], echo time (TE) 68 [ms], B-values 0 respectively 1000 [s/mm^2^], acquisition time (TA) 4:33 [min:s]), T2*-weighted gradient echo imaging (T2*w) (acquisition type 3D FLASH, first-order flow compensation in all three spatial directions, field of view 220 × 193 [mm^2^], number of slices 88, voxel size 0.9 × 0.9 × 1.6 [mm^3^], TR 27 [ms], TE 20 [ms], band width 120 [Hz/px], TA 3:29 [min:s]), fluid-attenuated inversion recovery (FLAIR) (acquisition type 3D, field of view 240 × 233 [mm^2^], number of slices 176, voxel size 0.5 × 0.5 × 1.0 [mm^3^], TR 4700 [ms], TE 386 [ms], inversion time (TI) 1530 [ms], TA 5:59 [min:s]), and T2-weighted imaging (T2w) (acquisition type 2D, turbo-spin echo approach (TSE), field of view 220 × 220 [mm^2^], number of slices 44, voxel size 0.4 × 0.4 × 3.0 [mm^3^], slice gap 0.3 [mm], TR 8180 [ms], TE 100 [ms], TA 3:26 [min:s]), sequences were acquired in transverse orientation.

Gradient-echo data were processed as outlined in detail below using in-house software that was written in Matlab (The Mathworks Inc., Natick, MA, USA) and utilized FSL’s brain extraction tool for brain masking^27^. Magnitude and phase images of the T2*w gradient-echo sequence were employed to compute susceptibility weighted images utilizing Laplacian-filtering as described in^28^. Quantitative susceptibility maps were computed from the phase images of the SWI scan by applying Laplacian-based phase unwrapping^29^ followed by “sophisticated harmonic artifact reduction for phase data with variable spherical kernels” (V-SHARP)^30^ to eliminate background fields and by “homogeneity enabled dipole inversion” (HEIDI)^31^ to solve the ill-posed inverse problem between local magnetic field and magnetic susceptibility. Residual non-harmonic background field contributions, e.g., resulting from B1-related transceiver phase were implicitly suppressed by applying V-SHARP with high-pass filter regularization (cut-off frequency = 0.012 mm^−1^)^32^. Susceptibility maps were referenced to the mean cerebrospinal fluid (CSF) susceptibility. To measure CSF susceptibility, masks were drawn bilaterally in the center of the anterior horn of the lateral ventricles in three axial slices as described in Straub et al.^33^. Susceptibility values are given in parts-per-million (ppm). Finally, maximum-intensity projections of susceptibility maps were calculated across a slab 12.8 mm thick.

### MR image analysis

**Figure 2:**
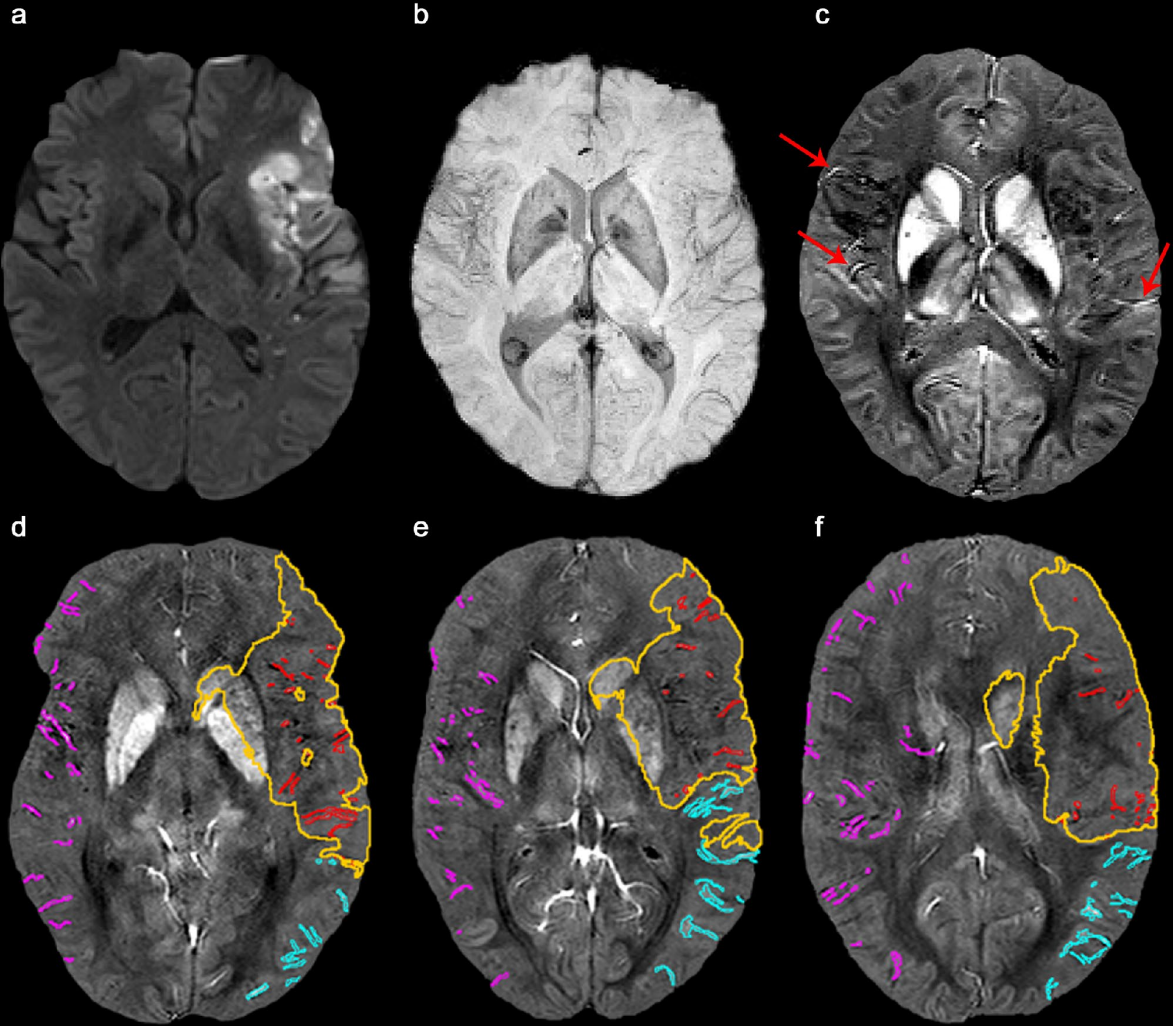
MR images of a stroke patient aged 49 years with successfully recanalized MCA-M1 occlusion on the left hemisphere. The DWI (**a**) shows the hyperintense infarct area on the left hemisphere. The SWI (**b**) helps identifying the localization of vessels. The corresponding susceptibility map is depicted in (**c**). Veins with higher levels of paramagnetic deoxyhemoglobin content appear brighter (red arrows). Representative susceptibility images covering the MCA territory are shown in (**d**–**f**). Cortical veins are delineated in different colors depending on the territory. Within the stroke area (yellow line) defined by DWI cortical veins are delineated in red, those within the ipsilateral non-ischemic MCA territory in cyan and those in the contralateral MCA territory in magenta.

**Figure 3:**
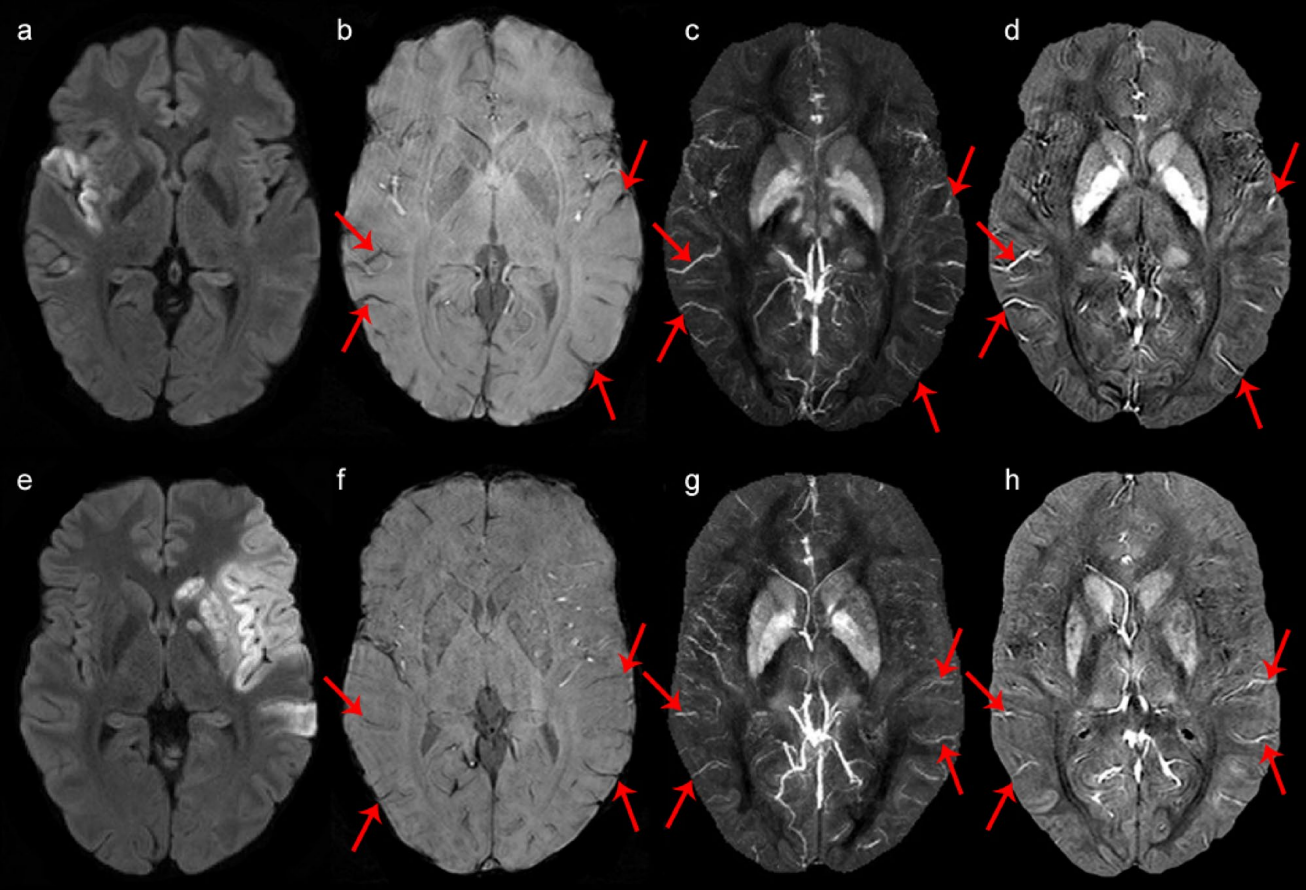
Comparison of DWI, SWI, MIP and QSM in 2 different patients for cortical vein identification. (**a**–**d**) represent one patient while (**e**–**h**) represent another patient. Image (**a**) and (**e**) demonstrate the ischemic core of the ischemic hemisphere, which is hyperintense on DWI. A selection of cortical veins (red arrows) is demonstrated on SWI (**b** and **f**), MIP (**c** and **g**) and QSM (**d** and **h**) in control and ischemic hemisphere. Note that the visibility of cortical veins within the infarction core is reduced. Cortical veins within the ipsilateral non-ischemic MCA territory and contralateral MCA territory seem to have comparable intensity.

In all patients, the same six slices representative for the MCA territory were selected. MCA territories were anatomically defined as areas supplied by the middle cerebral artery. Massive hemorrhages compromising cortical vein detection (applicable to three patients) were identified and excluded from the analysis considering standard imaging approaches for hemorrhages (SWI and CT scans were checked for each patient). Images were then analyzed using the Image J (NIH) software^34^. For each individual, a threshold was applied such that pixel above this threshold were considered to be veins without marking any background tissue. Thresholds were kept constant during analysis of each individual. Susceptibility values from QSM images within semi-automatically drawn masks of individual cortical veins that appeared prominent were analyzed, regardless of their site. An example is demonstrated in Fig. 2 showing the DWI, SWI and QSM as well as cortical veins on 3 representative slices of the MCA territory. When cortical veins were not identifiable semi-automatically because of interference with background tissue, a manual selection was added. In that case, we inspected the susceptibility maps and their maximum intensity projections (MIP) across 6 mm as well as the SWI image to identify cortical veins precisely. In addition, all semi-automatically drawn maps were visually compared to susceptibility maps, susceptibility weighted images and their MIPs to correct for contributions of uncompletely removed background fields originating from the sinus cavities and background tissue, which is shown in Fig. 3. Vein masks were drawn by two independent experts. Both examiners then agreed on what should be included into the vein mask. The interrater variability was analyzed utilizing Interclass Interrater Coefficient (ICC), which showed values between 0.94 and 0.98, indicating high interrater reliability (please see more detailed information in the supplementary material). Vein susceptibilities were calculated from the 10% highest signal within the vein mask to minimize partial volume effects; and values from both examiners were averaged. A distinction was made between veins within the stroke core, the ipsilateral non-ischemic MCA territory and the contralateral MCA territory. The stroke core was identified as hyperintense tissue signal on DWI and delineated semiautomatically utilizing the ImageJ threshold tool for delineation and manual correction with a consistent threshold for each individual. Thresholds for stroke core delineation were divergent between individuals.

### Statistical analyses

All statistical analyses were perfomed using the IBM SPSS Statistics software version 25. Comparisons between groups were done using the non-parametric Kruskal–Wallis-test. For correlation analyses between susceptibility values, clinical parameters (NIHSS on admission and mRS after 3–5 months), severity of ADC decrease within the infarction and infarct volume, we calculated Pearson’s correlation coefficient. A p-value below *0.05* was considered as statistically significant.

### Statement of ethics

The study was approved by the local ethics committee (Kantonale Ethikkomission Zurich; KEK-ZH-Nr 2014-0304). All procedures performed were in accordance with the ethical standards of the institutional and/or national research committee and with the 1964 Word Medical Association Declaration of Helsinki and its later amendments or comparable ethical standards. Informed consent was obtained from all participants as required by the ethics protocol.

## Results

### Patient cohort and clinical characteristics

During the period of December 2016 until October 2018, out of 263 patients with ischemic stroke referred for thrombectomy at the Department of Neurology, University Hospital Zurich, 23 patients with successfully recanalized MCA-M1/M2 occlusion were included in the study according to study plan.

Patients’ clinical characteristics are shown in Table 1. The median age of the patient population was 72 years. The most common risk factors were hypertension (57%) and atrial fibrillation (43%). Cardioembolic events were observed in more than 50% of the cases and therefore the most common cause of stroke in our patient population. In eight patients (35%) the cause of stroke could not be determined. Duplex sonography flow values of the recanalized MCA-M1/M2 were derived from patient charts and are shown in Table 2. In two cases, results from duplex sonography were not available. Flow in the ipsilateral MCA-M1 was similar to the contralateral side, confirming successful recanalization.

**Table 1:** Patient clinical characteristics.

	Stroke patients
	(n = 23)
**Demographic data**	
Age, median (IQR)	72 (45–90)
Female, *n (%)*	10 (43%)
**Risk factors, *n (%)***	
Diabetes mellitus	4 (17%)
Dyslipidemia	6 (26%)
Smoking	7 (30%)
Hypertension	13 (57%)
Coronary heart disease	2 (9%)
Atrial fibrillation	10 (43%)
Persistent foramen ovale	0 (0%)
**Stroke etiology, *n (%)***	
Large-vessel disease	1 (4%)
Cardioembolic	12 (52%)
Small-artery disease	0 (0%)
Other causes	2 (9%)
Undetermined	8 (35%)

Clinical characteristics of all 23 patients. Numbers (*n*) and percentage or median and interquartile range (*IQR*) are shown.

**Table 2:** Treatment and outcome characteristics.

	Duplex parameters
	(n = 21)
**Flow MCA-M1 [cm/s], median (IQR, min/max)**	
Ipsilateral systolic	97 (49, 47–178)
Contralateral systolic	94 (42, 60–146)
Ipsilateral diastolic	39 (20, 22–62)
Contralateral diastolic	37 (12, 20–61)

	Treatment parameters
	(n = 23)
**Clinical scores, median (IQR, min/max)**	
NIHSS on admission	13 (6, 3–19)
NIHSS after 24 h	5 (7.5, 0–22)
NIHSS improvement	4 (9.5, − 9–14)
mRS after 3 months	2 (2.5, 0–6)
Onset-to-needle time [min], median (IQR, min/max)	150 (111, 40–390)

Treatment and Outcome characteristics. In order to assess the reestablished blood flow, the ipsilateral and contralateral MCA-M1 were examined using duplex sonography. In 2/23 cases duplex sonography examination was not possible. Median, interquartile range (*IQR*), minimum and maximum are shown.

Information on neurological deficit upon admission and 3–5 months after discharge from the hospital is shown in Table 2. The median NIHSS on admission was 13 (IQR 6, Minimum 3, Maximum 19) and was improved by a median of 4 points (IQR 9.5, Minimum − 9, Maximum 14) on day 1 while the median mRS 3–5 months after hospitalization was 2 (IQR 2.5, Minimum 0, Maximum 6). 18/23 (78%) patients received intravenous thrombolysis in addition to thrombectomy. The median onset-to-needle time for patients receiving intravenous thrombolysis was 150 min (IQR of 111, Minimum 40, Maximum 390).

### Analysis of susceptibility values of cortical veins

Shown in Table 3, the median (IQR; Min–Max) infarct volume was 33.52 cm^3^ (71.5; 1.07–250.8). The median number of veins found within the stroke area was 9 (21; 2–88).

**Table 3:** QSM susceptibility and infarct size.

	Stroke patients
	(n = 23)
**QSM susceptibility cortical vein values, median (IQR, min/max)**	
Stroke area	0.050 (0.02, 0.03–0.08)
Ipsilateral infarct surrounding area	0.085 (0.04, 0.05–0.12)
Contralateral MCA territory	0.080 (0.03, 0.05–0.14)
Infarct size [cm^3^], median (IQR, min/max)	33.5 (71.5, 1.1–250.8)

QSM susceptibility values of cortical veins within the stroke area, the ipsilateral infarct surrounding MCA territory and the contralateral MCA territory. Additionally, we calculated the median infarct size on DWI. Median, interquartile range (*IQR*), minimum and maximum are shown.

**Figure 4:**
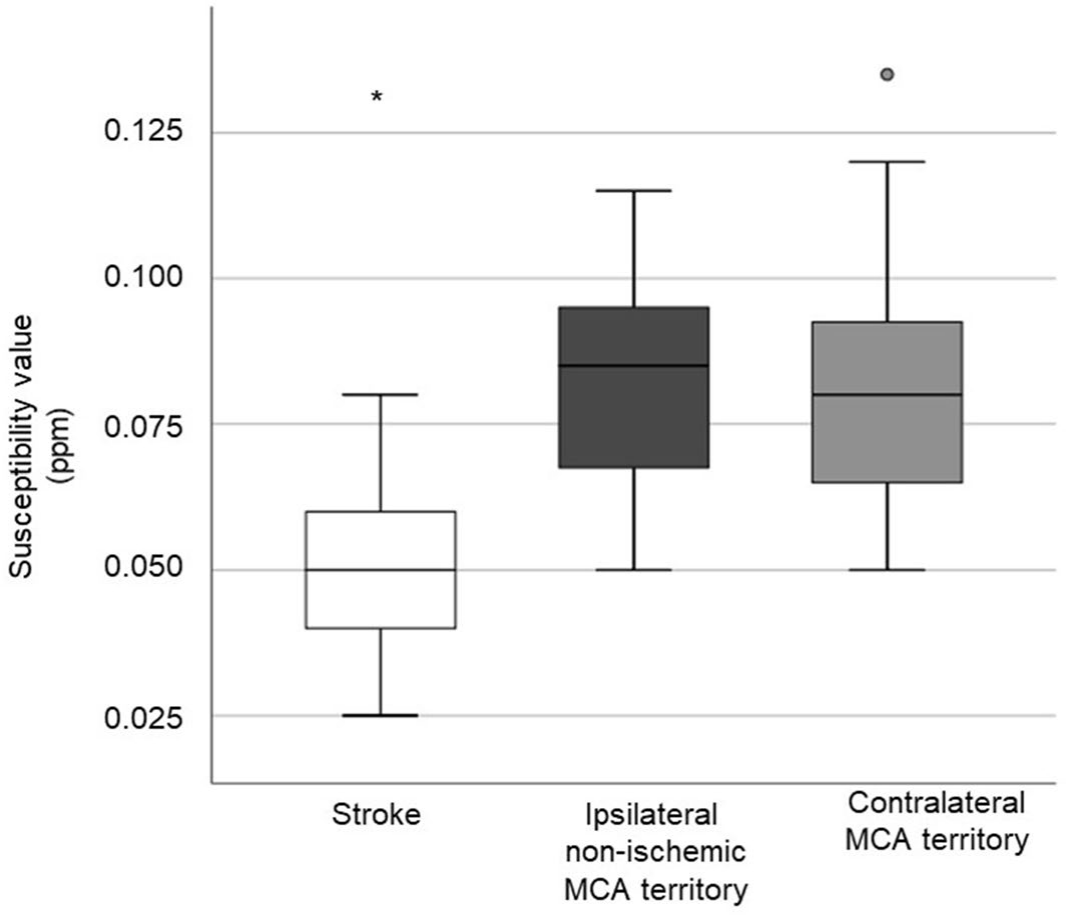
Comparison of the susceptibility values of the cortical veins within the stroke area, the ipsilateral non-ischemic MCA territory and the contralateral MCA territory. Areas that significantly (p < 0.05) differ from other areas regarding their susceptibility value are indicated (*)°indicates outliers.

In Fig. 4 and Table 3, the mean susceptibility values of cortical veins within the stroke core area, the ipsilateral non-ischemic MCA territory and the contralateral MCA territory of all stroke patients are shown. The median (IQR; Min–Max) susceptibility value of the cortical veins in the stroke areas was 0.050 ppm (0.02; 0.03–0.08). The median susceptibility value of the ipsilateral non-ischemic MCA territory was 0.085 ppm (0.04; 0.05–0.12) and of the contralateral MCA territory was 0.080 ppm (0.03; 0.05–0.14). The observed susceptibility values in cortical veins of the infarct area were 41% lower compared to the ipsilateral non-ischemic MCA territory and 38% lower compared to the contralateral MCA territory (p-value < 0.001). There was no significant differences in magnetic susceptibility between veins in the ipsilateral non-infarcted MCA territory and contralateral MCA territory.

Cortical vein susceptibilities within the infarct core did not correlate with early infarct volume (Pearson’s correlation coefficient of 0.276, p-value = 0.202) or severity of mean initial ADC decrease within the infarct (Pearson’s correlation coefficient of − 0.151, p-value = 0.493). We further analyzed a potential relationship between venous susceptibilities within the infarct and the clinical parameters initial stroke severity on NIHSS or 3–5 months functional disability on mRS, and found no such correlation (for NIHSS on admission: Pearson’s correlation coefficient of − 0.161, p-value = 0.464; for 3–5 month mRS: Pearson’s correlation coefficient of 0.253, p-value = 0.253).

## Discussion

In patients successfully recanalized by thrombectomy after stroke due to occlusion of the MCA-M1/M2 segment, we found distinct patterns of cortical vein susceptibility in the ipsilateral MCA territory using QSM. While lower venous susceptibility values were found within the infarct core, susceptibilites in the ipsilateral non-infarcted MCA territory were similar to the venous susceptibilities within the unaffected contralateral MCA territory.

QSM has been proven useful to asses vascular oxygenation in the brain^14^. Several papers have reported prominent veins (APCV) on susceptibility maps in patients with hyperacute and acute stroke, i.e. veins that were more visble and had larger diameter and length than those on the contralateral hemisphere or in healthy controls^18,21^. The presence of APCV has been hypothesized to be related to increased deoxyhemoglobin resulting from increased OEF^13,35^. A previous study found APCV in the infarct territories of hyperacute stroke patients (mean time from symptom onset to MRI acquisition 15 ± 4 h)^22^. The authors concluded that these prominent vessels could reflect a decrease of oxygen saturation in APCV which was estimated to be 16–44%. Luo et al. demonstrated that patients with APCVs have a tendency of deterioration of the neurological deficit while patients with no apparent APCVs tend to improve^18^. A study by Kim et al. has shown that APCV are not only observed in the cortex areas but also in deep thalamostriatal areas and that susceptibility values showed a strong inverse correlation with initial NIHSS^36^. It is expected that APCV disappear upon restoration of tissue perfusion, but this has not been investigated with QSM systematically.

We selected patients with verified recanalization of the MCA territory by catheter angiography (TICI 2b or TICI 3 score according to the TICI reperfusion grade) as well as duplex sonography measurements, which showed similar flow in the ipsi- and contralateral MCA-M1 segment. APCVs were clearly not detected in cortical regions. We found two vessel types within the affected vascular territory: veins with decreased QSM signal in the infarct core and veins with normal susceptibility values in the non-infarcted MCA territories. While we have not evaluated the presence of APCVs in the acute stage, one possibility is that the absence of prominent vessels represents a normalization of the OEF in the non-ischemic territories after reperfusion and oxygenation is restored^35^. Further studies are needed to investigate oxygenation status after acute ischemic stroke. Our results are in contrast to two recent preclinical QSM studies that detected increased QSM susceptibility values in veins within the infarct or penumbral areas after reperfusion^16,19^. A study observed increased venous susceptibilities after induction of a transient occlusion in the middle cerebral artery in rats. The increased venous susceptibilities were related to lower venous oxygen saturation^16^. A study in mice found higher susceptibility values in the ischemic hemisphere, particularly in the infarct border zone, after transient middle cerebral artery occlusion^19^. Immunohistochemistry revealed the presence of compressed capillaries and dilated larger vessels. This suggests that in animal models of transient cerebral ischemia, recanalization of the major feeding artery does not lead to a complete reperfusion of cerebral tissue and the appearance of prominent vessels with an increased OEF may serve compensatory purposes. Such no-reflow or reperfusion failure has been described in both animal models and stroke patients, representing a serious obstacle to recovery^37^.

DWI is an established imaging method to evaluate early infarct size and predict imaging as well as clinical outcome^38^. We did not find a correlation of susceptibility values to stroke severity, functional disability, or final infarct size in our patients. Therefore, we did not find evidence that susceptibility measured after recanalization in patients with stroke due to MCA-M1/M2 indicates severity of tissue damage.

The flow-compensated T2*-weighted data utilized for QSM were acquired in the clinical setting with the SWI acquisition scheme established in-house, providing a familiar view to the radiologists. Thus, venous susceptibility analysis relied on a single echo acquired with TE = 20 and a long echo readout and came without an additional measurement, as only the post-processing needed adjustment. Although multi-echo gradient-echo imaging has been suggested for QSM in particular for studying iron depositions, their application for studying venous vessels is not recommened, as 3D flow-compensation is typically not provided for all echoes. Incomplete flow compensation leads to flow-related phase contributions that are not due to magnetic susceptibility^39–42^. Consequently, non-local artifacts occurring adjacent to vessels with high blod flow might occur on the susceptibility map^40^.

### Limitations

As a limitation of our study, MRI measurements were done at a single time point, so that transient shifts in QSM signal earlier or later may have been missed. Furthermore, pathophysiological differences between stroke due to MCA occlusion in rodents and MCA stroke in human patients such as stroke duration/severity, collateral flow, or medication (anticoagulation, blood pressure medication) might play a role, causing a different dynamic of tissue oxygenation^35^. While venous ROIs were carefully identified based on MIP and SWI maps, the resulting venous susceptibility values are prone to partial volume effects, which is indicated by the low QSM values in small veins in our study. We calculated the venous susceptibility from the 10% highest signal within the vein mask to overcome the problem of partial volume effects.

Another limitation of our study is the lack of information about reperfusion status on capillary level. While results of catheter angiography and duplex sonography conducted in our patient cohort indicates full reperfusion, this is not comparable to an in-depth (e.g., immunohistochemical) confirmation about reperfusion on a capillary level. Since no perfusion imaging was performed at the time of QSM imaging, there was no assessment of tissue perfusion available to confirm reperfusion status. However, vessels in the peri-infarct region were mainly located in the area of hypoperfusion on the initial (pre-thrombectomy) perfusion images.

While we chose a homogenous patient cohort (all recanalized MCA-M1/M2 occlusions) to draw more valid conclusions according to the affected vascular territory and recanalization the sample size is small. Another limitation is that masks for individual cortical veins were drawn semi-automatically and some vessels might not have been identified because of edema compressing them. However, the semi-automatic approach allowed to carefully inspect and correct blood vessel segmentations. Finally, we did not compare our results to healthy controls, but used the contralateral hemisphere for comparison instead, which may have altered susceptibility as well.

## Conclusion

In conclusion, we found distinct patterns of cortical vein susceptibility after recanalization of acute ischemic stroke and our findings indicate that the susceptibility signal is highly dynamic in stroke. As QSM reflects tissue oxygenation in stroke patients with recanalized MCA-M1/M2 occlusion, it has potential to aid in understanding the complex processes that follow transient cerebral ischemia.

Further studies in larger patient cohorts on QSM vein susceptibilities may derive differences in stroke tissue prognosis with clinical implications for stroke patients.

## Supplementary Information

Supplementary Information.
