# Supplementary material for: Quantitative susceptibility mapping in ischemic stroke patients after successful recanalization
Source: Sci Rep. 2021 Aug 6;11:16038. doi: 10.1038/s41598-021-95265-3 (PMC8346586; doi:10.1038/s41598-021-95265-3)
Supplement: Supplementary file 1 — Supplementary Information. [file 41598_2021_95265_MOESM1_ESM.docx]

**Quantitative Susceptibility Mapping in Ischemic Stroke Patients after Successful Recanalization**

Jasmin Probst^1^, Marco Rohner^1^, Malin Zahn^1^, Marco Piccirelli^2^, Athina Pangalu^2^, Andreas Luft^1,5^, Andreas Deistung^3^, Jan Klohs^4^, Susanne Wegener^1*^

^1^ Deptartment of Neurology, Clinical Neuroscience Center, University Hospital Zurich and University of Zurich, Switzerland

^2^ Deptartment of Neuroradiology, Clinical Neuroscience Center, University Hospital Zurich, Switzerland

^3^ University Clinic and Outpatient Clinic for Radiology, University Hospital Halle (Saale), Germany

^4^ Institute for Biomedical Engineering, University of Zurich and ETH Zurich, Switzerland

^5^ Cereneo Center for Neurology and Rehabilitation, Switzerland

**Supplementary Information**

**Interclass Correlation Coefficient for cortical vein masks drawn independently by two experts.**

|  | Interclass Correlation Coefficient (ICC, 95% CI) |
| --- | --- |
| Stroke area | 0.968 (0.926-0.986) |
| Ipsilateral infarct surrounding area | 0.943 (0.864-0.976) |
| Contralateral MCA territory | 0.981 (0.956-0.992) |

ICC estimates and their 95% confident intervals were calculated using SPSS statistical package version 25.
